# Further Insights Into the Interaction of Human and Animal Complement Regulator Factor H With Viable Lyme Disease Spirochetes

**DOI:** 10.3389/fvets.2018.00346

**Published:** 2019-01-31

**Authors:** Jovana Jasmin Mühleip, Yi-Pin Lin, Peter Kraiczy

**Affiliations:** ^1^Institute of Medical Microbiology and Infection Control, University Hospital of Frankfurt, Frankfurt, Germany; ^2^Department of Biomedical Science, State University of New York at Albany, Albany, NY, United States; ^3^Division of Infectious Diseases, New York State Department of Health, Wadsworth Center, Albany, NY, United States

**Keywords:** *Borrelia*, complement, factor H, Lyme disease, immune evasion, spirochete, innate immunity, host-pathogen interaction

## Abstract

Spirochetes belonging to the *Borrelia (B.) burgdorferi* sensu lato (s.l.) complex differ in their ability to establish infection and to survive in diverse vertebrate hosts. Association with and adaption to various hosts most likely correlates with the spirochetes' ability to acquire complement regulator factor H (FH) to overcome the host's innate immune response. Here we assessed binding of serum FH from human and various animals including bovine, cat, chicken, dog, horse, mouse, rabbit, and rat to viable *B. burgdorferi* sensu stricto (s.s.), *B. afzelii, B. garinii, B. spielmanii, B. valaisiana*, and *B. lusitaniae*. Spirochetes ectopically producing CspA orthologs of *B. burgdorferi* s.s., *B. afzelii*, and *B. spielmanii*, CspZ, ErpC, and ErpP, respectively, were also investigated. Our comparative analysis using viable bacterial cells revealed a striking heterogeneity among Lyme disease spirochetes regarding their FH-binding patterns that almost mirrors the serum susceptibility of the respective borrelial genospecies. Moreover, native CspA from *B. burgdorferi* s.s., *B. afzelii*, and *B. spielmanii* as well as CspZ were identified as key ligands of FH from human, horse, and rat origin while ErpP appears to bind dog and mouse FH and to a lesser extent human FH. By contrast, ErpC did not bind FH from human as well as from animal origin. These findings indicate a strong restriction of distinct borrelial proteins toward binding of polymorphic FH of various vertebrate hosts.

## Introduction

Lyme disease (LD) is the most prevalent vector-borne anthropozoonosis in Eurasia and North America. This disease is caused by spirochetes belonging to the *Borrelia (B.) burgdorferi* sensu lato (s.l.) complex (1). The *B. burgdorferi* s.l. complex comprises more than 15 species including *B. burgdorferi* sensu stricto (*B. burgdorferi*), *B. garinii, B. afzelii, B. spielmanii, B. valaisiana*, and *B. bavariensis* (formally designated as *B. garinii* OspA serotype 4). Spirochetes are maintained in multiple vertebrate reservoir hosts (mainly mammals, birds, and reptiles) and transmitted from these hosts to humans and other animals during the blood meal of ixodid ticks (2). Upon the tick bite, spirochetes first survive in the blood, migrate from ticks to vertebrate hosts, and establish infection of the skin at the bite site (3). *B. burgdorferi* s.l. then disseminate via the bloodstream to multiple tissues and organs (1). In humans, the colonization of spirochetes can result in severe chronic infections such as Lyme arthritis, neuroborreliosis, or acrodermatitis chronica atrophicans (2, 4, 5). Thus, *B. burgdorferi* s.l. requires the ability to survive during the ticks' blood meal and in the hosts' bloodstream to be maintained in the enzootic cycle.

Complement is one of the most powerful innate immune defense mechanisms in vertebrate animals' blood. Complement is composed of a network of more than 50 proteins including inactive precursor molecules, fluid-phase, and membrane-bound regulators as well as distinct inhibitors (6–10). This tightly-controlled surveillance system plays an important role for the recognition, discrimination, and elimination of invading pathogens (7). Activation of complement is initiated through three canonical routes, the classical, the lectin, and the alternative pathways, all of which converge in the generation of the central C3b molecule and subsequently lead to the formation of the C3 and C5 convertases. Cleavage of C5 by the C5 convertases following binding of C5b to the microbial surface initiates the activation of the terminal sequence. Finally, a pore-forming complex known as the terminal complement complex (TCC) or membrane attack complex (MAC) is generated by the unidirectional, sequential binding of components C6, C7, and C8 to deposited C5b. This is followed by binding of numerous C9 molecules to the surface-associated C5b-8 complex. The integration of numerous pores into the cell membrane leads to the bacteriolysis of invading pathogens (9, 10). To prevent activated effector molecules from attacking self-cells and -tissues, this system is efficiently controlled at different levels by various soluble and membrane-anchored regulators (11). C1 esterase inhibitor (C1-INH) and the C4b-binding protein (C4BP) represent the main soluble regulators of the classical pathway while Factor H (FH) and Factor H-like protein 1 (FHL-1) are the primary regulators of the alternative pathway (11, 12). The latter two regulators act as co-factors for factor I-mediated inactivation of C3b, and thereby inhibit the formation and accelerate the decay of the C3 convertase of the alternative pathway (11, 13–15).

Recruitment of FH and FHL-1 appears to be an efficient and prominent strategy adopted by LD spirochetes to resist complement-mediated killing by termination of alternative pathway activation (16–19). *B. burgdorferi* s.l. produce at least five distinct surface-exposed Complement Regulator-Acquiring Surface Proteins (CRASPs), including CspA (CRASP-1), CspZ (CRASP-2), ErpP (CRASP-3), ErpC (CRASP-4), and ErpA (CRASP-5) [for review see (20, 21)]. The deficiency of CspA in infectious *B. burgdorferi* results in the inability to bind human FH (22). Conversely, the production of this protein in a spirochete strain leads to greater levels of human FH-binding activity (22, 23). Consistent with the unique expression of *cspA* when spirochetes are within ticks, this gene is essential for *B. burgdorferi* to be transmitted from nymphal ticks to mice by evading complement during ticks' blood meal (3). Unlike *cspA*, the other CRASP encoding genes are co-expressed when spirochetes are in vertebrate hosts (24). Though expressing each of these genes (except *erpC*) in spirochetes enhances human FH binding activity (20), a *cspZ* deletion mutant of *B. burgdorferi* strain B31 or Tn-inserted mutant spirochete of *erpP* or *erpA* display little or no defect of human FH-binding activity and/or infectivity, suggesting a potential redundant function of these genes *in vitro* and *in vivo* (25). In addition to *B. burgdorferi*, previous studies on other *B. burgdorferi* s.l. species revealed that the overall FH binding pattern often resembles the pattern of serum resistance/susceptibility observed among LD spirochetes corroborating the hypothesis of a species-specific, complement-associated host selectivity (26–28). Taking up this important issue, numerous attempts have been made to determine the ability of certain spirochetal proteins to bind to serum-derived FH from different non-human vertebrate animals including mouse, rat, cat, dog, sheep, horse, cattle, goat, monkey, mini pig, pig, duck, quail, and chicken (3, 20, 29–38) and to further substantiate the hypothesis that complement is an important factor for host-specificity and transmissibility of LD spirochetes. These previous investigations were conducted to detect binding of polymorphic FH to recombinant proteins or to cell lysates by Far western blotting. However, controversial results have been reported from these studies, possibly due to the usage of misfolded or denatured proteins (borrelial cell lysates or purified proteins) for capturing serum-derived FH (29–37). In this study, we thus re-assessed binding of native FH from different animals' sera to viable *B. burgdorferi* s.l. as well as gain-of-function borrelial strains producing each of the CRASPs to overcome the obvious limitations of previous approaches. Such a strategy allowed us to definitely delineate the CRASP-mediated FH-binding activity in a fashion that is much closer to physiological conditions.

## Materials and Methods

### Ethics Statement

The study and the respective consent documents were approved by the University Hospital Frankfurt' ethics committee (control number 160/10). All healthy blood donors provided written informed consent.

### Bacterial Strains and Culture Conditions

Borrelial strains listed in Supplementary Table 1 as well as transformed spirochetes listed in Supplementary Table 2 were grown until mid-exponential phase (5 × 10^7^ cells per ml) at 33°C in Barbour-Stoenner-Kelly (BSK-H) medium (Bio&SELL, Feucht, Germany) or BSK-H supplemented with streptomycin (50 mg/ml) as described previously (39). The density of spirochetes was determined using dark-field microscopy and a Kova counting chamber (Hycor Biomedical, Garden Grove, CA).

### Human and Animal Sera, Antibodies, and Serum Proteins

Normal human serum (NHS) used for serum susceptibility testing and Far-Western blotting or as a source of FH was tested for the presence of anti-*Borrelia* IgM and IgG antibodies by commercially available ELISAs (Enzygnost® Borreliosis/IgM and Enzygnost® Lyme link VlsE/IgG, Siemens Healthcare Diagnostics Products GmbH, Marburg, Germany). Only sera proven to be negative for IgM and IgG anti-*Borrelia* antibodies were used to form a serum pool of at least 10 blood donors. Chicken, goat, bovine, mouse, rat, and sheep serum were purchased from Dunn Labortechnik, Asbach, Germany. Cat and dog (beagle) serum was purchased from Sera Laboratories International, West Sussex, UK, and horse and rabbit serum were obtained from Sigma-Aldrich, Taufkirchen, Germany. Purified human FH was purchased from Complement Technology, Tyler, TX, USA, and the polyclonal sheep anti-FH antiserum (dilution 1/1,000) was obtained from Acris, Herford, Germany.

### Binding of Complement Proteins to Viable Spirochetes

Spirochetes (2 × 10^9^ cells) grown to mid-log phase were gently washed and resuspended in 750 μl human or animal serum supplemented with 34 mM EDTA (pH 8.0) to avoid complement activation. After 1 h incubation at room temperature and four wash steps with PBSA (0.15 M NaCl, 0.03 M phosphate, 0.02% sodium azide, pH 7.2) containing 0.05% Tween-20, proteins bound to the spirochetal surface were eluted by incubation with 100 mM glycine-HCl (pH 2.0) for 15 min. Cells were then removed by centrifugation at 14,000 x g for 10 min at 4°C, and the supernatant was neutralized by adding 1M Tris (pH 9.0). Both, the last wash and the eluate fraction were separated by 12.5% glycine-SDS-PAGE under non-reducing conditions and binding of human or animal FH was analyzed by Western blotting using a sheep anti-FH antibody (dilution 1:500). Detection of bound proteins was performed using 3,3′,5,5′-tetramethylbenzidine (TMB) as substrate.

### Far-Western Blot Analysis

Whole cell lysates obtained from each borrelial isolate (15 μg per lane) were subjected to 10% tris/tricine SDS-PAGE under reducing conditions and transferred to nitrocellulose as previously described (40–42). Briefly, membranes incubated for 1 h with NHS were washed four times with TBS containing 0.2% Tween 20 and thereafter incubated for 1 h with a sheep anti-FH antiserum (dilution 1:1,000). Following four wash steps with TBS containing 0.2% Tween 20, membranes were incubated with an appropriate peroxidase-conjugated secondary antibody for 1 h. Detection of bound proteins was performed using TMB as substrate.

### Production of His_6_-Tagged Borrelial Proteins in *E. coli*

Generation of vectors pQE CspA LW2, pQE CspA PKo, pQE CspA A14S, pQE CspZ, pBLS538, and pBLS528 producing amino-terminally polyhistidine-tagged CspA_LW2_ (CRASP-1 of *B. burgdorferi* s.s. strain LW2), CspA_PKo_ (CRASP-1 of *B. afzelii* strain PKo), CspA_A14S_ (CRASP-1 of *B. spielmanii* strain A14S), CspZ (CRASP-2), ErpP (CRASP-3), and ErpC (CRASP-4), respectively were described previously (39, 43–45). Note that CRASP-3/ErpP and CRASP-4/ErpC encoded by the *erpP* and *erpC* genes of *B. burgdorferi* s.s. type strain B31 and the European *B. burgdorferi* s.s. strain LW2 are identical and thus, proteins are hereafter referred to as CspA_B31_, ErpP, and ErpC. Expression of recombinant proteins was induced in DH5α or JM109 at an OD_600_ of 0.6 by the addition of 0.2 mM IPTG. Following incubation for 4 h at room temperature, cells were centrifuged (5,000 × g, 20 min, 4°C) and subsequently suspended in lysis buffer (300 mM NaCl, 56 mM NaH_2_PO_4_ pH 8, 10 mM Imidazole) containing 50 mg/ml lysozyme. Cells were lysed by 6 rounds of sonication for 30 s using a Branson B-12 sonifier (Heinemann, Schwäbisch Gmünd, Germany). After centrifugation (14,000 × g, 20 min, 4°C), supernatants were filtered through 0.45 μm filters and stored at −20°C until needed. For affinity purification of His-tagged proteins, cell lysates were incubated with Ni-NTA agarose and bound proteins were purified as recommended by the manufacturer (QIAGEN, Hilden, Germany).

### Generation of CRASP Producing *B. garinii* Cells

The construction of shuttle vectors pCspA, pCspA_PKo_, pCspA_A14S_, pCspZ, pErpP, pErpC allowing ectopical production of surface exposed lipoproteins in the surrogate strain *B. garinii* G1 and the procedure applied to generate transformed spirochete have been previously described in detail (39, 41, 44, 45). The functional characterization of the transformed strains including G1/pCspA, G1/pCspA_PKo_, G1/pCspA_A14S_, G1/pCspZ, G1/pErpP, and G1/pErpC has been described previously (39, 41, 44, 45).

### Detection of Interacting Serum Proteins Using Magnetic Beads

For identification of interacting serum proteins, magnetic beads (Dynabeads TALON, Invitrogen Dynal AS, Oslo, Norway) coated with cobalt ions were used. Briefly, His_6_-tagged proteins (10 μg each) were allowed to bind to Dynabeads (Life Technologies) for 10 min at room temperature as recommended by the manufacturer. Following several wash steps, recombinant proteins coated to magnetic beads were incubated with 500 μl serum for 1 h at room temperature. After four washes with phosphate buffer (50 mM phosphate, 300 mM NaCl, 0.01% Tween 20), bound proteins were eluted with elution buffer (300 mM imidazol, 50 mM phosphate, 300 mM NaCl, 0,01% Tween 20) for 15 min at room temperature. The eluate and the last wash fraction were separated by 12.5% glycine SDS-PAGE under non-reducing conditions followed by silver staining.

### Mass Spectrometry

The selected proteins were excised from the acrylamide gels and analyzed by mass spectrometry using a commercial service. Data generated were analyzed by Mascot Search Engine, Matrix Science (http://www.matrixscience.com) and the peptides identified are summarized in Supplementary Table 3. The protein bands present in the eluate fraction but absent in both wash and control fractions (uncoated beads) were then applied the in-gel digestion. The resulting peptides were analyzed using mass spectrometry. The obtained spectra were analyzed using the NCBI.fasta protein database, and results yielding a score of >80 were considered confident (*p* < 0.05).

## Results

### Detection of Polymorphic FH in Animal Sera

**Figure 1:**
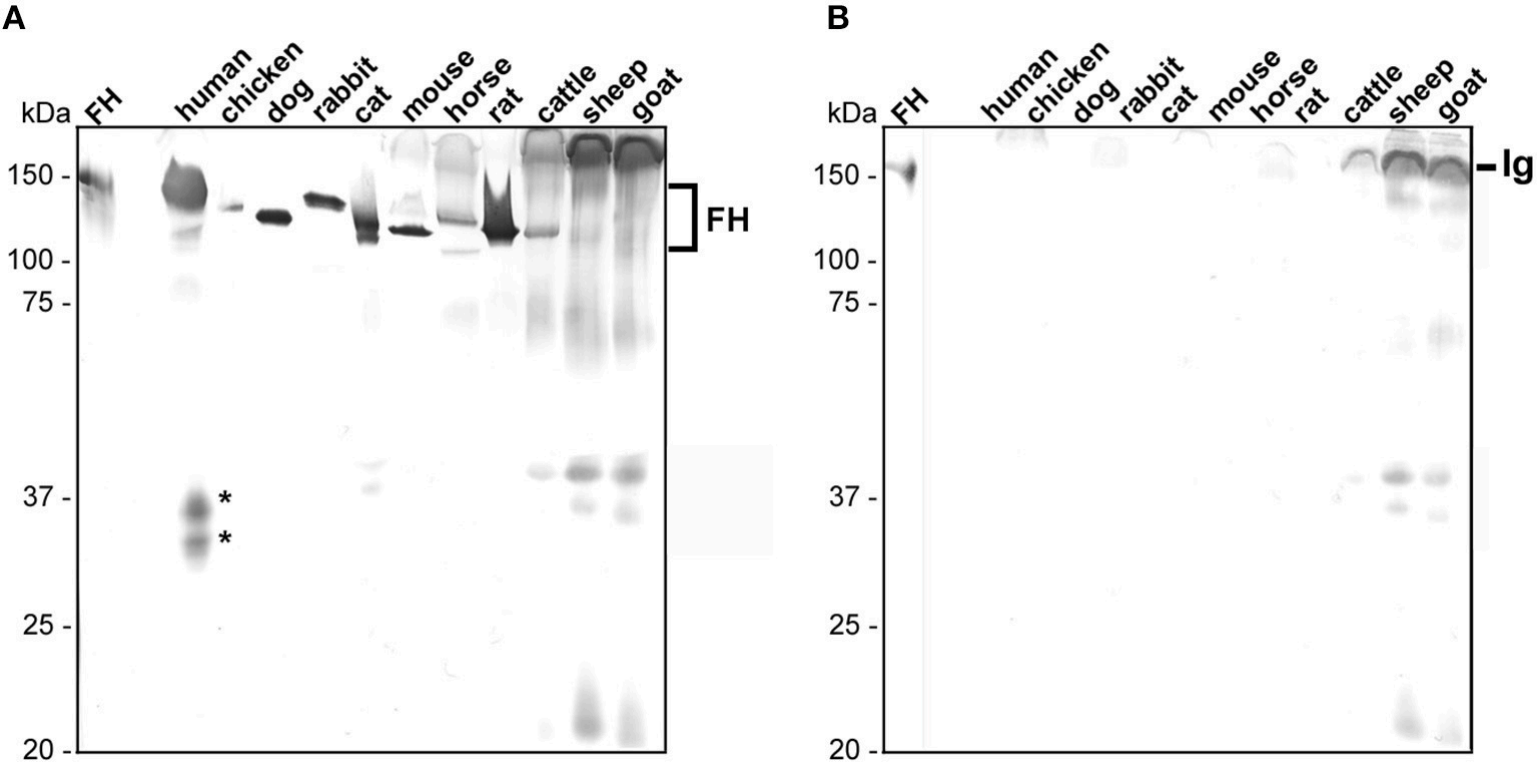
Detection of polymorphic FH in animal sera. Equal amounts of sera of different origins were loaded to two 12.5% glycine-SDS gels and separated serum proteins were transferred to nitrocellulose. **(A)** Human and animal FH were detected using a sheep anti-FH serum (dilution 1:500) and a secondary anti-sheep antibody (1:1,000). **(B)** Cross-reactivity of the secondary anti-sheep antibody to polymorphic FH molecules (dilution 1:1,000). Purified human FH (500 ng) at the left of **(A,B)** was used as control. Human FHR proteins are indicated by asterisks and cross-reacting immunoglobulins (Ig) from cattle, sheep, and goat are specified. The mobility of the molecular mass standard is indicated on the left.

In order to detect native FH in human serum as well as polymorphic FH from rodents (mouse, rat), carnivores (cat, dog), lagomorphs (rabbit), ungulates (horse, cattle, goat, sheep), and bird (chicken), we first searched for a suitable antibody, exhibiting a broad reactivity to FH molecules of various origin. Of note, serum samples from birds other than chicken as well as from lizards were not commercially available in sufficient quantity at the time of this study. As various monoclonal and polyclonal antibodies tested failed to recognize FH from the selected samples (data not shown), a polyclonal sheep anti-FH antibody raised against the human FH reacted with FH from nearly every animal's serum except with FH from sheep and goat (Figure 1A; FH, ~150 kDa). As expected, sera from the two latter animals showed a strong reactivity to immunoglobulins and generated a strong background in the Western blot analysis. A strong reactivity could be detected for FH from human, mouse, rat, cat, dog as well as rabbit whereby a weaker reactivity was observed for chicken, horse, and cattle. In addition, proteins of 25 to 40 kDa could be detected in sera obtained from human, cat, cattle, sheep, and goat suggesting cross-reactivity of this antibody to factor H-related proteins (FHRs). An additional signal of ~110 kDa could be observed in sera derived from human, cat, and cattle. The nature of these proteins exhibiting a lower molecular mass is at yet unknown and could be partial denatured FH molecules, polymorphic variants of FH or other cross-reacting molecules. For the comparative analysis, only signals of 150 kDa were considered. Furthermore, by analyzing cross-reactivity of the secondary anti-sheep antiserum, immunoglobulins from cattle, sheep, and goat showed the strongest signal while immunoglobulins from human displayed a very weak cross-reactivity. None of the other sera tested reacted with the secondary antibody applied (Figure 1B). Owing to the broad reactivity of the sheep anti-FH antibody to almost all FH molecules, we used this particular antibody for all subsequent analyses.

### FH Binding to the Surface of Viable Spirochetes

In order to analyze FH binding to spirochetes in a close-to-native context, we first employed whole-cell ELISA by immobilizing 5 × 10^7^ borrelial cells per well. However, several attempts (e.g., optimization of the blocking buffer formulation, variation of the antibody, and cell concentration) failed to enhance the signal-to-noise ratio for all animal sera tested which yielded very low absorbance values (<0.2) after subtracting the background values (data not shown). Because of the limitations and failure to increase the sensitivity of the whole-cell ELISA, e.g., by coating higher amounts of spirochetes, we decided to re-assess FH binding by using a fluid phase approach where high concentrations of viable spirochetes (2 × 10^9^ cells) were incubated in inactivated sera including human, mouse, rat, cat, dog, rabbit, cattle, horse, and chicken. Since the sheep anti-human FH antiserum did not reliably detect FH from goat and sheep, these sera were not included in these analyses. Following serum incubation, bound FH was eluted from the surface of the spirochetes and the last wash and the eluate fraction were subjected to glycine-SDS-PAGE under non-reducing conditions. Polymorphic FH molecules were then detected by Western blotting using the sheep anti-human FH antibody. Purified human FH was applied in all Western blot analysis as a positive control.

**Figure 2:**
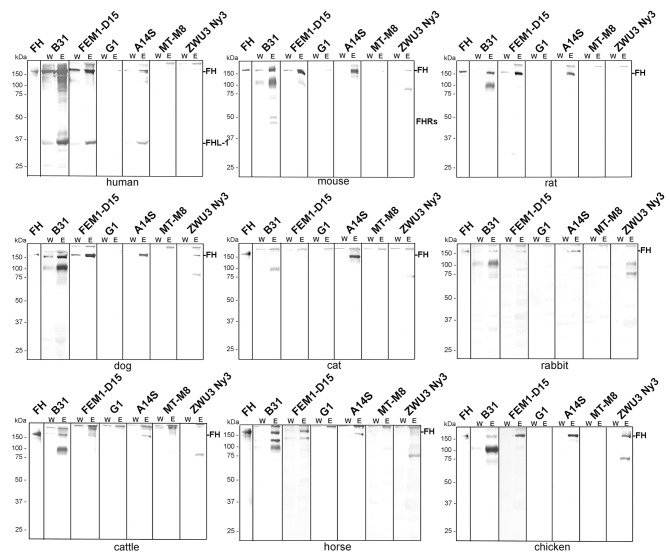
Binding of polymorphic FH to viable LD spirochetes. Indicated *Borrelia* strains were incubated in human serum as well as in mouse, rat, dog, cat, rabbit, cattle, horse, or chicken serum. Following serum incubation, spirochetes were washed extensively, and surface-bound proteins were eluted using 0.1 M glycine (pH 2.0). Both, the last wash (W) and the eluate (E) fractions obtained from each reaction were separated by glycine-SDS-PAGE under non-reducing conditions and transferred to nitrocellulose. FH molecules were detected by using a sheep anti-FH antibody (1:500). Purified human FH (500 ng) served as a control in all tests. The mobility of the molecular mass standard is indicated on the left of each panel.

As expected, *B. burgdorferi* B31, *B. afzelii* FEM1-D15, and *B. spielmanii* A14S bound **human** FH as well as FHL-1 while *B. garinii* G1, *B. lusitaniae* MT-M8, and *B. valaisiana* ZWU3 Ny3 did not bind human FH at all (Figure 2) (46–48). Concerning *B. burgdorferi* B31, there were four hardly detectable signals below 37 kDa which most likely represent FH-related proteins FHR-1α, FHR-1β, FHR-2, and FHR-2α (44, 49).

Employing **mouse**, **rat**, or **dog** serum, strong signals corresponding to the molecular weight of human FH could be detected in *B. burgdorferi* B31, *B. afzelii* FEM1-D15, and *B. spielmanii* A14S (Figure 2). In addition, two faint bands of ~150 and 75 kDa were present in *B. valaisiana* ZWU3 Ny3 when mouse or dog serum was applied. Notably, *B. garinii* G1 and *B. lusitaniae* MT-M8 did not bind FH molecules from any of the animal sera examined. Apparently, none of the borrelial strains bound FH from **cat** with the exception of *B. spielmanii* A14S where a strong signal was visible. **Rabbit** serum showed only very weak signals corresponding to human FH when *B. burgdorferi* B31, *B. afzelii*

FEM1-D15, and *B. spielmanii* A14S were applied while additional weak signals were observed for *B. valaisiana* ZWU3 Ny3. By incubating spirochetes with serum from **cattle** and **horse**, a stronger signal was only detected for *B. burgdorferi* B31 whereas weak signals were also seen in *B. afzelii* FEM1-D15 and *B. spielmanii* A14S. Moreover, an additional band of ~120 kDa and two bands of 90 and 120 kDa were observed when *B. burgdorferi* B31 was treated with cattle and horse serum, respectively. Concerning *B. valaisiana* ZWU3 Ny3, several signals of weak intensity below 100 kDa could be observed. Concerning **chicken** serum, strong reactive bands corresponding to the molecular weight of human FH was observed for *B. afzelii* FEM1-D15, *B. spielmanii* A14S, and *B. valaisiana* ZWU3 Ny3 while a weak signal was visible when employing *B. burgdorferi* B31. In the eluate of the latter strain, a 100 kDa-protein exhibited a strong signal as well as a 75 kDa-protein in *B. valaisiana* ZWU3 Ny3. The data collected from the Western blot analyses are summarized in Table 1.

**Table 1:** Binding of polymorphic FH to diverse borrelial species.

**Species**	**Human**	**Mouse**	**Rat**	**Cat**	**Dog**	**Rabbit**	**Cattle**	**Horse**	**Chicken**
Bb^a^ B31	+	+	+	–	+	±	±	+	±
Ba FEM1-D15	+	+	+	–	+	±	±	±	+
Bg G1	–	–	–	–	–	–	–	–	–
Bs A14S	±	+	+	+	+	±	±	±	+
Bl MT-M8	–	–	–	–	–	–	–	–	–
Bv ZWU3 Ny3	–	±	–	–	±	–	–	–	±

a *Bb, B. burgdorferi; Ba, B. afzelii; Bg, B. garinii; Bs, B. spielmanii; Bl, B. lusitaniae; Bv, B. valaisiana*.

*+, strong signal; ±, weak signal; –, no signal detectable*.

### Binding of Polymorphic FH to CRASP-Producing Spirochetes

**Figure 3:**
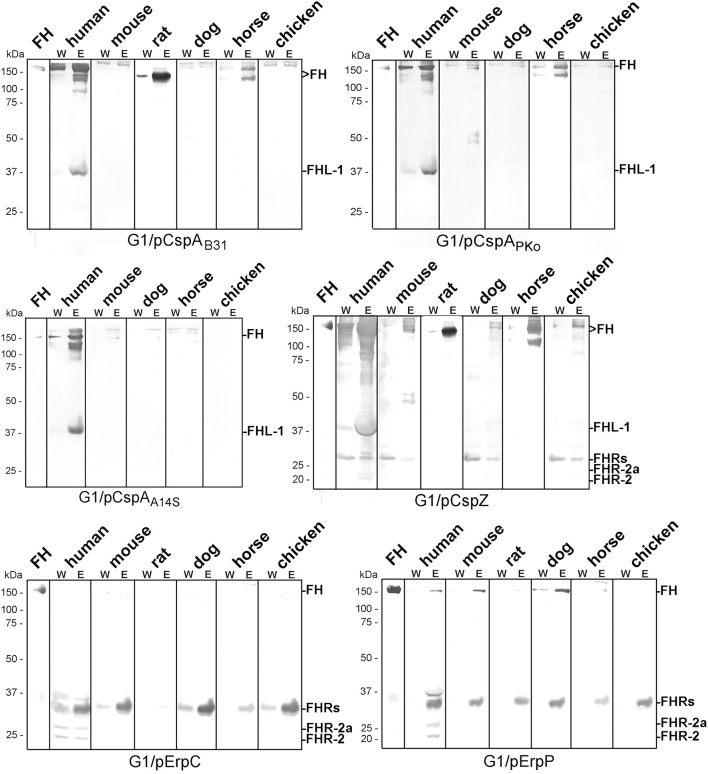
Identification of CRASP involved in binding of polymorphic FH molecules. *Borrelia* strains ectopically producing CspA_B31_, CspA_PKo_, CspA_A14S_, CspZ, ErpC, or ErpP were incubated in human serum and various animal sera. Following incubation, spirochetes were washed extensively and surface-bound proteins were eluted using 0.1 M glycine (pH 2.0). Both, the last wash (W) and the eluate (E) fractions obtained from each reaction were separated by glycine-SDS-PAGE under non-reducing conditions and transferred to nitrocellulose. FH molecules were detected by using a sheep anti-FH antibody (1:500). Purified human FH (500 ng) served as a control. The mobility of the molecular mass standard is indicated on the left of each panel.

To further explore the role of CRASPs in binding polymorphic FH molecules, *B. garinii* strain G1 producing CspA from *B. burgdorferi* B31, CspZ, ErpC, or ErpP were incubated with human serum and selected animal sera. ErpA was not included as this protein displayed similar binding patterns as ErpP (29, 41, 44). Transformants producing CspA orthologs from *B. afzelii* PKo (CspA_PKo_) or *B. spielmanii* A14S (CspA_A14S_) were also included as these CspA orthologs were documented to promote binding to human FH and FHL-1 and also confer resistance to complement-mediated killing when grown in 50% of human serum (45). However, differences in the capability between CspA_B31_ and CspA_PKo_ to bind purified horse FH have previously reported (3). As shown in Figure 3, all three CspA orthologs acquired human FH as expected. CspA_B31_ and CspA_PKo_ also bound to horse FH whereas CspA_B31_ also bound to rat FH. None of the CspA orthologs interacted with FH from mouse, dog, and chicken serum. Regarding CspZ, human, rat, and horse serum showed the strongest signals and weak signals were observed for mouse, dog, and chicken serum. Weak signals were visible when human, mouse, and dog serum was applied to the ErpP-producing strain. Notably, the ErpC-producing strain was unable to bind FH from any of the animal sera investigated. The data collected from these analyses are summarized in Table 2.

**Table 2:** Binding of polymorphic FH to diverse CRASP.

**Species**	**Human**	**Mouse**	**Rat**	**Dog**	**Horse**	**Chicken**
CspA_B31_	+	–	–	–	+	–
CspA_PKo_	+	–	nd	–	+	–
CspA_A14S_	+	–	nd	–	–	–
CspZ	+	±	+	±	+	±
ErpC	–	–	–	–	–	–
ErpP	±	±	–	+	–	–

*+, strong signal; ±, weak signal; –, no signal*.

*nd, not determined*.

Direct comparison of the pattern obtained from the CRASP-producing transformants with that gather from the wild-type strains revealed that in most instances CRASP proteins are responsible for the acquisition of serum-derived FH (Supplementary Figure 2). CspA orthologs from *B. burgdorferi* B31, *B. afzelii* PKo or FEM1-D15, and *B. spielmanii* A14S were primarily responsible for the binding of human FH and FHL-1 as well as horse FH. CspZ like CspA strongly bound human FH and FHL-1 (39, 50) as well as horse FH. Hardly visible bands at the size of human FH could also be observed employing mouse, dog, and chicken serum. In addition, CspZ bound an additional protein of 120 kDa derived from horse serum. ErpC did not bind FH at all whereby ErpP seemed to bind FH from human, mouse, and dog. However, cross-reactive bands below 37 kDa were only visible when CspZ, ErpC, and ErpP-producing strains were investigated but these signals were absent in the wild-type strains (see Figure 2). Therefore, it is tempting to speculate whether these signals correspond to polymorphic FHR proteins of the animal sera analyzed. Concerning ErpC and ErpP, it has previously been shown that both proteins display a strong interaction with human FHR-1α, FHR-1β, FHR-2, and FHR-2a (41, 44). Thus, binding of FHRs to the CRASP-producing strains cannot be completely excluded and further investigations are required to definitively identify these interacting proteins.

### Identification of CRASP-Interacting Serum Proteins

**Figure 4:**
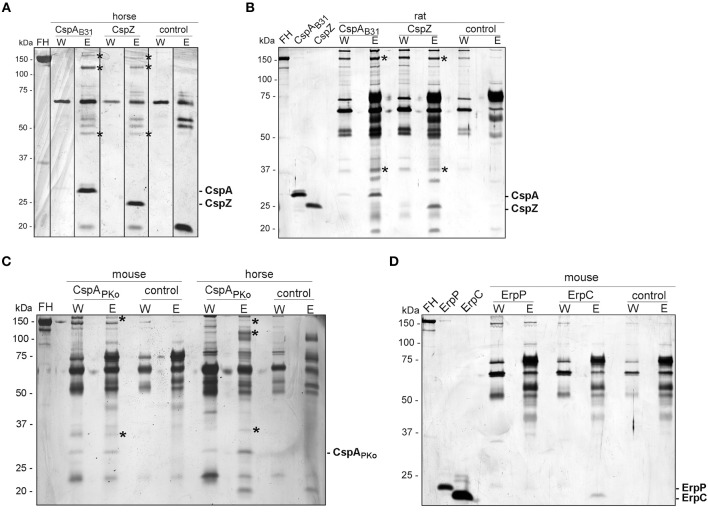
Identification of FH molecules bound to CRASP proteins. Purified, polyhistidine-tagged proteins (10 μg each) coupled onto magnetic beads were incubated with selected animal sera. Immobilized CspA_B31_ and CspZ were incubated with horse **(A)** or rat **(B)** serum, CspA_PKo_ with mouse or horse serum **(C)** and ErpP and ErpC with mouse serum **(D)**. Uncoated beads were also incubated under the same conditions and used as a control to identify non-specific binding of serum proteins. After extensive washing, bound proteins were eluted with 100 mM glycine and the eluate fractions were separated by glycine-SDS-PAGE, following silver staining. Protein bands indicated were cored from stained gels and proteins were identified by mass spectrometry. The mobility of the molecular mass standard is indicated on the left of each panel and recombinant proteins eluted were indicated on the right. The asterisks in panel A, B, and C indicated the protein bands analyzed by mass spectrometry.

A pull-down assay was conducted to identify further serum proteins interacting with CspA, CspZ, ErpC, and ErpC (41). His-tagged CspA, CspZ, ErpC, and ErpC were coupled to magnetic beads and after incubation with the respective serum samples, bound proteins along with the immobilized borrelial proteins were eluted. Following separation of the eluates by SDS-PAGE, proteins were visualized by silver staining (Figure 4).

For the horse serum incubated with CspA and CspZ-coupled beads, three bands of 150-, 120-, and 45-kDa could be detected in the eluate fraction but not in the wash or control fractions (Figure 4A). The 120-kDa band represents horse FH and yielded a high mascot score of 456.56 (gi|338722817) (see Supplementary Table 3). The 45-kDa band was identified as a histidine-rich glycoprotein of *Equus cabalus* with a mascot score of 125.18 (gi|338715996). However, the 150-kDa band detected in horse serum did not yield any results. Compared to the control, two additional proteins of 150- and 37-kDa were found in rat serum bound to CspA and CspZ, but we were unable to identify these proteins using mass spectrometry as scores of these proteins were very low (below 80; Figure 4B).

The incubation of CspA_PKo_ with mouse serum yielded two proteins of 150- and 29-kDa whereas the interaction of this protein with horse serum gave three proteins of 150-, 120-, and 29-kDa (Figure 4C). Regrettably, all attempts to identify these proteins by mass spectrometry failed because all scores were lower than the threshold (80). We also treated magnetic beads coated with ErpC and ErpP with mouse serum, but no obvious differences in the protein patterns were detected between the ErpC/ErpP-immobilized and the uncoated control beads (Figure 4D).

## Discussion

Vector-transmitted pathogens, especially borreliae have developed a range of subtle strategies to infect, disseminate, and persist in their natural reservoir hosts. Different outer surface proteins act in concert to enable spirochetes to overcome the mammalian innate immune system, in particular the destructive effects of complement. One elegant way used by certain spirochetes is the recruitment of host-derived complement regulators such as FH to inhibit and breakdown complement at central activation points (17, 21, 51, 52). In this study, we re-assess the concept of a species-specific, FH-associated host selectivity of LD spirochetes (27, 28) by investigating bacterial cells challenged with human and various animal sera in a close-to-native context. In agreement with previous studies, the data presented herein also revealed that the FH binding patterns are highly consistent with the serum resistance phenotypes of the respective genospecies. Moreover, we demonstrated that both CspA and CspZ constitute the primary ligands for polymorphic FH molecules as recently demonstrated for the selective binding pattern of CspA orthologs of *B. burgdorferi, B. afzelii*, and *B. garinii* to mouse, horse or quail FH (3).

Previous studies on the interaction of FH with different genospecies are largely based on *in vitro* experiments where either whole borrelial cell lysates, purified borrelial proteins or the respective serum samples were subjected to SDS-PAGE following immobilization of the separated proteins to a solid membrane (29, 31, 32, 34, 35). These investigations revealed obvious discrepancies between the FH binding patterns obtained by different experimental approaches. For example, binding of FH from human and mouse but not from rat, cat, dog, cattle, horse, and guinea pig was found if cell lysates of *B. burgdorferi* strain SKT2 were applied (34) while in another study where purified borrelial proteins of *B. burgdorferi* strain B31 were investigated, binding of FH from human, mouse, rat, cat, dog, cattle, rabbit, cattle, horse, and goat could be detected (29). In a reverse setting where serum samples were separated and immobilized FH was used for capturing of selected borrelial proteins (see below), strong signals in the range of human FH were visible in the sera from mouse, dog, cow, monkey, mini pig, guinea pig, and pig but not in the sera from rat, cat, rabbit, horse, goat, duck, and chicken (31, 32, 53). Although, Far-Western blotting has been proven as a valuable methodology for detecting protein-protein interactions (54), the utilization of denatured or partially misfolded proteins for capturing may often lead to false-positive results as recently demonstrated for the interaction of human FH with ErpA, ErpC, and ErpP (41, 44). To overcome the technical drawbacks of those immunoassays, we incubated viable spirochetes in native but complement-inactive serum. In contrast to a previous study employing ELISA (19), we were able to utilize considerably greater amounts of spirochetes (2 × 10^9^ cells) and serum (750 μl) to increase the sensitivity in order to detect even minute amounts of cell-bound FH. Because specific antibodies recognizing FH molecules of different animals are currently not available, we used a sheep anti-human FH antibody that exhibited a broad species-specificity and yielded a low signal-to-noise ratio to the most relevant sera included in this study except sheep, cattle, and goat (Figure 1).

In addition, our comparative analysis revealed binding of proteins at the size of human FH and additional serum proteins >150 kDa (Figure 2). Assuming that the bands at the size of 150 kDa corresponded to the polymorphic FH molecules, *B. burgdorferi, B. afzelii*, and *B. spielmanii* bound FH derived from human, mouse, rat, and dog origin. Both, *B. afzelii* and *B. spielmanii* interacted with chicken FH while *B. spielmanii* was the only genospecies that acquired FH derived from cat serum. Binding of horse FH was detected for *B. burgdorferi* and weak signals were also found when assessing *B. afzelii* (Table 1). Of note, the 120-kDa band was confirmed by mass spectrometry to represent horse FH but not the 150-kDa band. *B. burgdorferi, B. afzelii*, and *B. spielmanii* appeared to bind rabbit FH to a lesser extent as the signals obtained were faint. A different binding pattern was observed for *B. valaisiana* ZWU3 Ny3 as only chicken serum gave a strong signal whereas weak signals were obtained for mouse and dog. Finally, *B. garinii* G1 (OspA serotype 6) and *B. lusitaniae* MT-M8 did not bind FH at all. Initial studies unveiled that host complement is an important factor for tick-to-host transmission, host specificity, and niche-adaptation, and therefore for the global ecology of LD spirochetes (26, 27, 55–57). Our results, thus strongly support the previous findings of a species-specific host adaption by which the main complement regulator of the alternative pathway FH is a key tie-in.

The second objective of our study was the identification of the surface-exposed determinants capturing serum-derived polymorphic FH molecules. In contrast to former investigations employing purified proteins or whole cell lysates as ligands (19, 31, 32, 34, 58), we aimed at analyzing spirochetes producing either native CspA (CRASP-1, BBA68), CspA orthologs from *B. afzelii* PKo and *B. spielmanii* A14S, CspZ (CRASP-2, BBH06), ErpP (CRASP-3, BBN38), or ErpC (CRASP-4) (Supplementary Figure 1) on the cell surface. As wild-type *B. garinii* G1 did not bind FH molecules at all (Figure 1), the signals obtained are attributed to the CRASP produced in the transformed strains (see Supplementary Figure 2 for direct comparison of the FH binding pattern of wild-type and CRASP-producing strains). In this study, we observed that spirochetes producing CspA_B31_ and CspA_Pko_ acquired FH and FHL-1 from human serum and FH from mouse serum, which is consistent with previous studies (59). Similarly, we found that CspA_PKo_ was able to interact with FH from horse serum (Figure 2 and Table 2). Note that recombinant CspA_PKo_ or this protein produced on the surface of a *cspA*-deficient *B. burgdorferi* strain is incapable of binding to purified horse FH (3). These results imply that the horse FH may have to be in the serum to bind to CspA_PKo_. Additionally, CspA_B31_ was previously shown to be incapable of binding to mouse and horse FH using Far-Western blotting (32). As the structure of immobilized FH may be altered or even destroyed, this finding suggests the essentiality of FH's native structure for CspA to display binding activity. Furthermore, consistent with previous studies showing broad spectrum of interaction with various hosts' FH (53), we found that CspZ binds to FH from human, rat, dog, and horse as well as mouse, dog, and to a lesser extent chicken (Figure 2 and Table 2). These findings suggest that both CspA and CspZ play a role in evading different hosts' complement. Binding of FH by CspA and CspZ, which are produced while spirochetes are within the ticks and within the vertebrate hosts, respectively, raises the possibility that these determinants facilitate survival and persistence of LD spirochetes in these distinct and hostile environments (3, 60).

In contrast to ErpC, which failed to exhibit any binding capability for human and animal FH, ErpP appeared to strongly bind FH derived from dog serum but also displayed weak binding to human and mouse FH (Figure 3 and Supplementary Figure 2). Although, binding of murine FH has been shown previously using solid-phase immunoassays with purified proteins (18, 30, 33, 34) others investigators did not detect binding of Erp orthologs to mouse FH (31). In addition, potential FH-binding proteins belonging to the Erp protein family have been detected in *B. afzelii, B. garinii, B. bavariensis, B. valaisiana*, and *B. andersonii* which seems to bind FH derived from mice, rats, dogs, or cats (34). These proteins might also contribute to the pathogenesis of LD spirochetes.

## Conclusion

In closing, the overall FH binding patterns as demonstrated in the present study largely resembles the pattern of serum susceptibility observed among LD spirochetes corroborating the concept of species-specific, complement-associated host selectivity.

## Author Contributions

JM: study design, experimental work, data interpretation, figure preparation, and final approval; Y-PL: data interpretation, drafting of the manuscript, and final approval; PK: study design, data interpretation, figures and table preparation, drafting of the article, and final approval.

### Conflict of Interest Statement

The authors declare that the research was conducted in the absence of any commercial or financial relationships that could be construed as a potential conflict of interest.
