# Supplementary material for: Further Insights Into the Interaction of Human and Animal Complement Regulator Factor H With Viable Lyme Disease Spirochetes
Source: Front Vet Sci. 2019 Jan 31;5:346. doi: 10.3389/fvets.2018.00346 (PMC6365980; doi:10.3389/fvets.2018.00346)
Supplement: Supplementary file 1 [file Image_1.pdf]

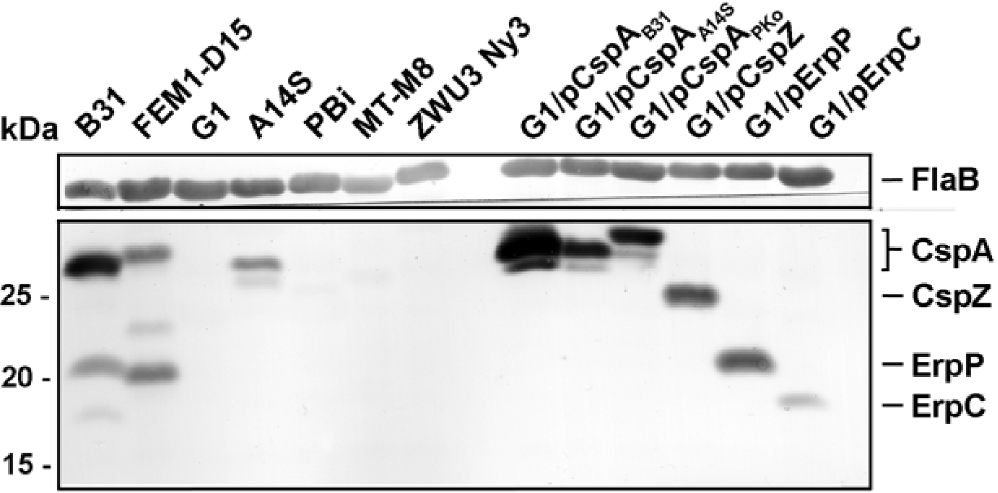

Supplementary figure 1. Identification of FH-binding proteins among borrelial strains by Far Western blotting. Cell lysates (30 µg each) obtained from wild type borrelial strains *B. burgdorferi* B31, *B. afzelii* FEM1-D15, *B. garinii* G1, *B. spielmanii* A14S, *B.bavariensis* PBi, *B. lusitaniae* MT-M8, and *B. valaisiana* ZWU3 Ny3 as well as from transformants producing distinct CRASP proteins were subjected to 10 % tris/tricine SDS-PAGE and transferred to nitrocellulose. The membrane was incubated with human serum as source for FH and potential FH-binding proteins were detected using a goat anti-FH serum (1:1,000). Monoclonal antibody L41 1C11 recognizing the FlaB protein was used to show equal loading of bacterial lysates. The CRASP proteins CspA, CspZ, ErpP, and ErpC of *B. burgdorferi* B31 are indicated on the right and the mobility of the marker proteins is indicated on the left.
