# Supplementary material for: Further Insights Into the Interaction of Human and Animal Complement Regulator Factor H With Viable Lyme Disease Spirochetes
Source: Front Vet Sci. 2019 Jan 31;5:346. doi: 10.3389/fvets.2018.00346 (PMC6365980; doi:10.3389/fvets.2018.00346)
Supplement: Supplementary file 2 [file Image_2.pdf]

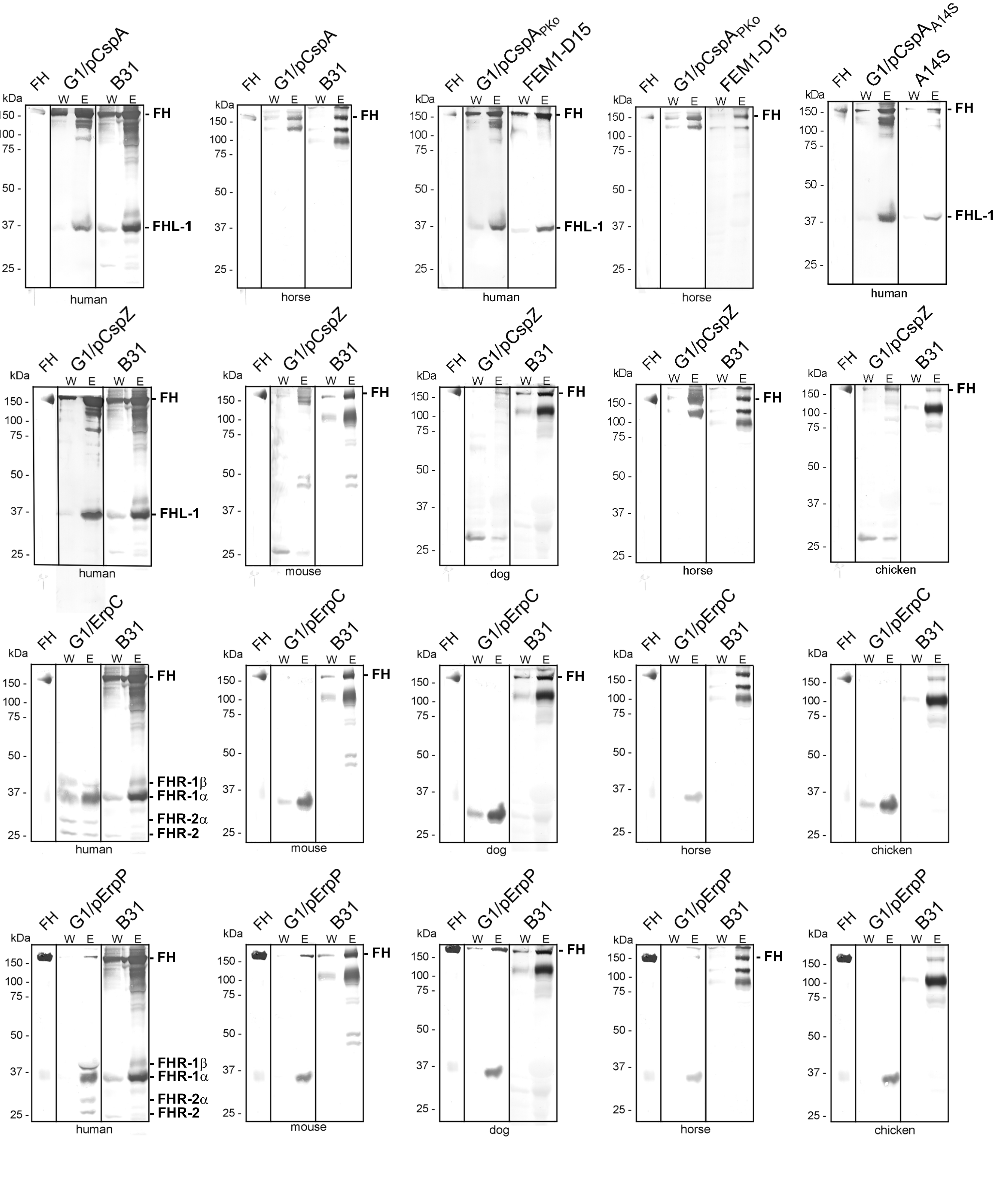

Supplementary figure 2. Determination of FH-binding proteins among *Borrelia* strains.

Selected membranes obtained from the Far Western blotting with the wild type *Borrelia* strains (see figure 2) as well as with transformants producing distinct CRASP proteins (see figure 3) were arranged next to each other for direct comparison.
