# Supplementary material for: Further Insights Into the Interaction of Human and Animal Complement Regulator Factor H With Viable Lyme Disease Spirochetes
Source: Front Vet Sci. 2019 Jan 31;5:346. doi: 10.3389/fvets.2018.00346 (PMC6365980; doi:10.3389/fvets.2018.00346)
Supplement: Supplementary file 3 [file Table_1.pdf]

Supplementary table 1. Borrelial strains used in the study

Genospecies	Strain	Origin		Serum susceptibility[a]
		Biological	Geographical	
B. burgdorferi	B31	Tick, *I. scapularis*	USA	Resistant
B. afzelii	FEM1-D15	Human, skin	Germany	Resistant
B. garinii	G1	Human, CSF[b]	Germany	Sensitive
B. spielmanii	A14S	Human, skin	The Netherlands	Resistant
B. lusitaniae	MT-M8	Tick, *I. ricinus*	Portugal	Sensitive
B. valaisiana	ZWU3 Ny3	Tick, *I. ricinus*	Germany	Resistant

[a]Serum resistance was determined by incubation of spirochetes in the presence of 50% NHS by a colorimetric growth inhibition assay and by immunofluorescence staining of deposited complement components C3, C6, and C5b-9 (Kraiczy et al., 2000)

[b]CSF, cerebrospinal fluid
