# Supplementary material for: Further Insights Into the Interaction of Human and Animal Complement Regulator Factor H With Viable Lyme Disease Spirochetes
Source: Front Vet Sci. 2019 Jan 31;5:346. doi: 10.3389/fvets.2018.00346 (PMC6365980; doi:10.3389/fvets.2018.00346)
Supplement: Supplementary file 4 [file Table_2.pdf]

Supplementary table 2. Borrelial transformants used in the study

Genospecies	Strain	Ectopically expressed protein	Strain description
B. garinii	G1	CspA (CRASP-1) from *B. burgdorferi* LW2	G1/ pCspA
B. garinii	G1	CspA (CRASP-1) from *B. spielmanii* A14S	G1/pCspA A14S
B. garinii	G1	CspA (CRASP-1) from *B. afzelii* PKo	G1/pCspA PKo
B. garinii	G1	CspZ (CRASP-2) from *B. burgdorferi* LW2	G1/pCspZ
B. garinii	G1	ErpP (CRASP-3) from *B. burgdorferi* LW2	G1/pErpP
B. garinii	G1	ErpC (CRASP-4) from *B. burgdorferi* LW2	G1/pErpC
