# Supplementary material for: Further Insights Into the Interaction of Human and Animal Complement Regulator Factor H With Viable Lyme Disease Spirochetes
Source: Front Vet Sci. 2019 Jan 31;5:346. doi: 10.3389/fvets.2018.00346 (PMC6365980; doi:10.3389/fvets.2018.00346)
Supplement: Supplementary file 5 [file Table_3.pdf]

Supplementary table 3. Data report of the peptide mass fingerprint search by MASCOT

Data report for the 120-kDa band of *Equus cabalus* corresponding to FH

Scores	Position of amino acids	Sequence	Modification
37.3	66-75	R.TLGTITMQCR.N	Carbamidomethyl: 9
58.9	76-86	R.NGQWVALNPSR.I	
35.8	154-164	K.CLPVTEPENGR.L	Carbamidomethyl: 1
23.3	248-254	R.GFEYSER.G	
40.5	262-271	K.FGWSPIPSCR.E	Carbamidomethyl: 9
26.9	384-395	R.AEGWSPAVPCLR.Q	Carbamidomethyl: 10
44.4	413-421	R.TYLQGESVK.V	
51.2	516-526	K.YCDMPVFENAR.A	Carbamidomethyl: 2
25.9	576-584	R.ECKIPQIER.Y	Carbamidomethyl: 2
27.2	579-584	K.IPQIER.Y	

Data for the 45-kDa band of *Equus cabalus* corresponding to a histidine-rich glycoprotein

Scores	Position of amino acids	Sequence	Modification
61.8	99-109	R.RPSDIVIGQCK.V	Carbamidomethyl: 10
63.4	171-179	R.ENDDFASFR.V	
